# Biofilm Consumption and Variable Diet Composition of Western Sandpipers (*Calidris mauri*) during Migratory Stopover

**DOI:** 10.1371/journal.pone.0124164

**Published:** 2015-04-14

**Authors:** Catherine B. Jardine, Alexander L. Bond, Peter J. A. Davidson, Robert W. Butler, Tomohiro Kuwae

**Affiliations:** 1 Bird Studies Canada, Pacific Wildlife Research Centre, Delta, British Columbia, Canada; 2 Department of Biology, University of Saskatchewan, Saskatoon, Saskatchewan, Canada; 3 Coastal and Estuarine Environment Research Group, Port, and Airport Research Institute, Yokosuka, Japan; Scottish Association for Marine Science, UNITED KINGDOM

## Abstract

Many shorebird species undertake long-distance migrations punctuated by brief stays at food-rich, estuarine stopover locations. Understanding use of these food resources helps guide conservation and responsible development decisions. We determined the extent and degree to which Western Sandpiper (*Calidris mauri*) utilized biofilm as a food resource across a large and variable stopover location during northward (spring) migration. We investigated the spatial heterogeneity in diet composition, to determine whether shorebirds were consistently feeding on biofilm or whether diet varied between naturally and anthropogenically delineated sites. We used stable isotope analysis to estimate that biofilm conservatively comprised 22% to 53% of Western Sandpiper droppings across all sampling sites and that prey composition differed significantly between areas within the stopover location. Widespread biofilm consumption demonstrates the importance of biofilm as a dietary component. Variable diet composition suggests that habitat heterogeneity may be an important component of high quality stopover locations in the context of “state-dependant trade-offs” of Western Sandpiper population sub-groups. Future management decisions must consider and address potential impacts on the biofilm community throughout a stopover location, as single site studies of diet composition may not be adequate to develop effective management strategies for entire stopover sites.

## Introduction

High-quality stopover locations are integral in the life cycle of many migratory birds [1–3]. Successful migration of gregarious shorebirds depends on their ability to acquire adequate resources from these locations, often over only a few days, to fuel long-distance migrations [4–5]. It is therefore important to understand how shorebirds use the food resources at stopover locations [6–7] to make appropriate conservation and land-use management decisions.

Intertidal mud and sandflats often host matrix-enclosed microorganism communities generically known as biofilm [8–9]. Biofilm is complex and variable but generally consists of a thin layer of organic detritus, microbes, meiofauna, and sediment which forms on the surface of intertidal flats. Biofilm is largely dominated by diatom species, in addition to microbes, prokaryotes, and eukaryotes suspended in a mucilaginous matrix of extracellular polymeric substances combined with non-carbohydrate organic compounds [8–10]. The density and quality of biofilm determine its potential as a food source; both of these factors vary spatially and temporally [7, 11–14]. Biofilm is a substantial food source for calidrid sandpipers at several migratory stopover sites [10, 13, 15–16].

Major estuarine stopover locations supporting biofilm are often strategic places for transport, industrial and residential development. Such development can significantly change the extent and quality of estuarine ecosystems by altering hydrology and sedimentation, converting habitats, and introducing pollutants or invasive species. Each of these factors could potentially affect biofilm quality, abundance and distribution. An understanding of the extent and degree to which migratory shorebirds use biofilm is therefore necessary, to assess the possible impacts of development, and to help guide design and management.

A globally significant portion of the world’s Western Sandpiper (*Calidris mauri*) population and the entire population of the Pacific subspecies of Dunlin (*Calidris alpina pacifica*) migrate along North America’s Pacific flyway, regularly stopping during spring northward migration at a few, food-rich locations that are separated by hundreds or thousands of kilometres [17–19]. The Fraser Estuary—Boundary Bay system in southwestern British Columbia, Canada is one of the larger stopover locations on this flyway, and is a globally significant Important Bird Area [20]. It is also located adjacent to a major urban center, supports a major port, and is subject to large-scale development proposals. We focus on Western Sandpipers because they are strongly associated with biofilm feeding [13] and are dependent on this stopover location; 14%–21% of the total flyway population of Western Sandpipers regularly passes through a small sub-section of the estuary during spring migration, possibly up to 42%–64% in some years [20–21]; when the additional extent of the estuary is taken into consideration, these proportions are likely much higher.

Western Sandpiper and Dunlin are widespread across the Fraser Estuary—Boundary Bay system during northward migration [22–23] but the relative density of birds varies throughout the area. Habitat choice theory for the Western Sandpiper is largely focused on the concept of “state-dependant trade-offs” [24–26], wherein different individuals may exhibit different dietary preferences. Factors driving site selection include predation risk [27–28], morphological sex differences such as variation in bill length [29], physiological condition [25, 30] and food availability [31–33, 28]. Biofilm comprises a substantial portion of Western Sandpiper diet during spring migration in a small section of the Fraser Estuary—Boundary Bay system [10, 13], yet the importance of biofilm and other prey to Western Sandpipers across the entire stopover location has not been studied, nor has the spatial heterogeneity in biofilm feeding and diet composition within the broader estuarine system been explored. Given that biofilm represents a rich, highly available food resource to which Western Sandpiper morphology is adapted [8, 13, 34–36] we expect biofilm feeding to be a strategy used throughout the stopover location. However, the multiple state-dependant trade-off hypotheses that have been proposed for this species, and the variation of bird density in the different areas of the stopover location, lead to the hypothesis that diet composition may vary between areas within the larger Fraser Estuary—Boundary Bay system.

Here we investigate the extent to which Western Sandpipers consume biofilm in different areas within the Fraser Estuary—Boundary Bay estuarine system and the spatial heterogeneity of their diet composition, to determine whether shorebirds consistently consume biofilm throughout the stopover location, and whether heterogeneity in diet composition occurs.

## Materials and Methods

**Fig 1:**
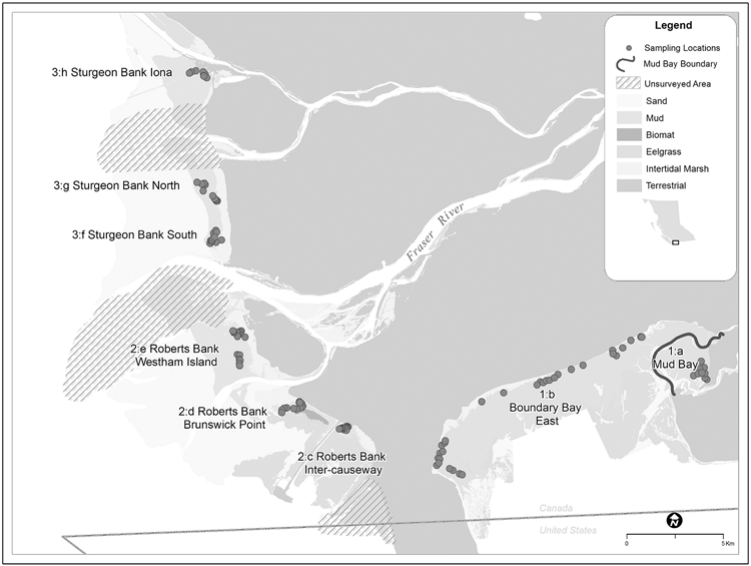
Distribution of sampling locations across the Fraser Estuary—Boundary Bay migratory stopover location, 23 April–7 May 2012.

Our study was conducted in three Wildlife Management Areas [37–39] of the Fraser Estuary—Boundary Bay system; 1—Boundary Bay (49.07, -122.96), 2—Roberts Bank (49.06, -123.16) and 3—Sturgeon Bank (49.16, -123.21). A total of eight sites within these three areas were used as sampling units (Fig 1). Two sites were located in Area 1:Boundary Bay (Site 1:a Mud Bay and Site 1:b Boundary Bay East), three sites in Area 2:Roberts Bank (Site 2:c Inter-causeway, Site 2:d Brunswick Point and Site 2:e Westham Island) and three sites were located in Area 3:Sturgeon Bank (Site 3:f Sturgeon South, Site 3:g Sturgeon North and Site 3:h Sturgeon Iona). Sites in the Fraser Estuary were separated by plumes of the Fraser River, and/or man-made jetties extending up to 2.5 km from shore, except Sturgeon Bank North and Sturgeon Bank South, which have different sediment and wave exposure characteristics. Mud Bay was delineated from Boundary Bay East by the Serpentine River channel; Mud Bay exhibits distinctly different physical and biological characteristics to Boundary Bay East. For the purposes of this study the term “stopover location” will refer to the entire Fraser Estuary—Boundary Bay system, the term “area” will refer to the three Wildlife Management Areas that make up the location (labeled 1 to 3) and the term “site” will refer to the eight naturally or anthropogenically delineated sampling sites that fall within the three Wildlife Management Areas (labeled a through h). This research was undertaken for the federal-provincial environmental assessment review for a new aviation fuel facility installation and overseen by the B.C. Environmental Assessment Office and Port Metro Vancouver. All sites were accessed via public routes, with the exception of Westham Island, which was accessed with permission from George C. Reifel Migratory Bird Sanctuary.

### Diet Composition

We sampled the diet of Western Sandpipers across the entire stopover location. We followed the approach used by Kuwae et al. [10], and modeled the isotopic values carbon (δ^13^C) and nitrogen (δ^15^N) in shorebird droppings and prey items to determine the relative contribution of each prey type to the overall diet [10, 40]. Droppings were chosen, rather than tissue or blood samples, as the best indicator of Western Sandpiper diet at the sample sites due to the short turnover time necessary for isotopic studies during migration stopover [4, 13]. Droppings unequivocally reflect the birds’ diets during the preceding hours and do not present the challenges inherent in interpreting fractionation [13]. The high polysaccharide and organic matter content in biofilm makes it highly digestible [8, 13, 34–36] and therefore the majority is assimilated into the body; hence droppings provide a conservative estimate of biofilm contribution to the overall diet [13]. Prey items were selected based on previous studies of stomach contents and feeding ecology of Western Sandpipers and were grouped by trophic level for a total of 3 prey groups including biofilm in the form of surface sediment, small invertebrates (primarily amphipods, cumaceans, bivalves and gastropods between approximately 1mm and 10mm in length) and polychaetes [10, 15, 41–42].

We focused on spring rather than fall migration in the Fraser Estuary—Boundary Bay system because the density of Western Sandpipers is higher and the period of migration shorter (two weeks, compared to two months in fall) [21, 23], presenting the opportunity to investigate the importance of diet composition and heterogeneity. Samples were collected between April 23 and May 7, 2012, from flocks comprising a minimum of 75% Western Sandpipers. Samples were collected within three hours following the first high tide of the day. Western Sandpiper droppings (n = 125) were collected directly from the intertidal mudflat surface from flocks feeding up to 1 km from the shoreline for at least 30 minutes prior to sample collection; no vertebrates were captured or disturbed during this study and no animal care approval was required. Western Sandpiper droppings were distinguished from those of Dunlin based on size and diameter [43]. Extreme care was taken that no surface sediments were included in the dropping samples; droppings that were disturbed or located in standing water were avoided. The top of each dropping was carefully scraped off while the portion in contact with the mudflat was left behind. For each sample ten or more droppings from different individuals were collected in a single 2 ml vial from within an area of approximately 50 m^2^, in order to gather a sufficient amount of material for isotope analysis. Surface sediment samples (n = 118) were collected from the same locations, using a toothbrush to scrape off the uppermost 1 mm of surface sediment [13]. Macroinvertebrates were collected by digging to a depth of 5cm and passing the substrate through a 1 mm mesh sieve, individuals greater than approximately 10 mm in length were discarded as they fell outside the size range expected to be consumed by Western Sandpipers, all species of non-polychaete invertebrates collected from each excavated sample were combined as one sampling unit for isotope analysis (n = 18). Large polychaetes were collected using the same method, digging to a maximum depth of 45cm (n = 12). Although this depth is outside the range accessible by Western Sandpiper these species are vertically mobile and retreat when disturbed, therefore deeper digging was required to capture them. Microphytobenthos samples (n = 16) were extracted from surface sediments following the methods of Kuwae et al. [10]. Sediment samples were spread in a tray to a depth of approximately 5 mm and a 60 μm mesh nylon screen was placed over the sediment followed by a layer of pre-combusted glass wool (450°C for 2 hours). The glass wool was moistened by spraying with filtered seawater (filtered using 60 μm mesh) and left overnight at ambient temperature (20°C) in the dark to allow for microphytobenthos migration into the glass wool. All samples were frozen at -20°C after collection.

#### Stable Isotope Analysis

Dropping samples were treated prior to stable isotope analysis to remove potential metabolites such as urea and ammonium. A 5 mg subsample of each powdered dropping sample was mixed with a 1.4 ml 2:1 chloroform:methanol solution. Particulate matter was allowed to settle and the solution was removed using a pipette. This procedure was repeated a minimum of three times per sample followed by oven drying.

Due to the high sediment content in samples collected from the intertidal zone, all samples were treated to remove non-dietary carbonates prior to isotope analysis using an acid wash [44]. A few drops of 1 mol/L HCl were added to each sample in a glass vial and samples were left overnight to allow full decomposition of carbonates. Samples were then placed on a hotplate at 50°C to remove any remaining HCl [45].

All samples were weighed into tin capsules and combusted in either a Carlo Erba NC2500 (dropping and invertebrate samples) or a Costech 4010 Elemental Analyzer (sediment and microphytobenthos samples) and delivered to mass spectrometers via continuous flow systems (Conflo II or Conflo III) using helium as a carrier gas. Stable-isotope values of carbon (*δ* ^13^C) and nitrogen (*δ* ^15^N), percent carbon content, and percent nitrogen content were measured using either a Thermo-Finnigan Delta Plus mass spectrometer (droppings and invertebrates) or a Delta XP isotope-ratio mass spectrometer (sediment and microphytobenthos) interfaced to an Elemental Analyzer via the Conflo II or Conflo III, respectively. Isotope values are presented relative to international measurement standards Vienna Peedee Belemnite (VPDB) and atmospheric nitrogen for *δ* ^13^C and *δ* ^15^N, respectively. Stable-isotope values are expressed in *δ* notation as deviation from the appropriate international measurement standard in parts per thousand (‰).

A variety of secondary isotopic reference materials (SIRMs) were used to ensure that values used in calibration spanned the range of expected values, and composition of unknowns. These showed that instrument precision was 0.2 for *δ* ^13^C, and 0.2 for *δ* ^15^N (S1 Table). In addition, 31/308, or 10% of samples were run in duplicate yielding a mean SD of 0.1–0.2 ‰ for *δ* ^13^C and *δ* ^15^N depending on sample type.

#### Microphytobenthos

**Fig 2:**
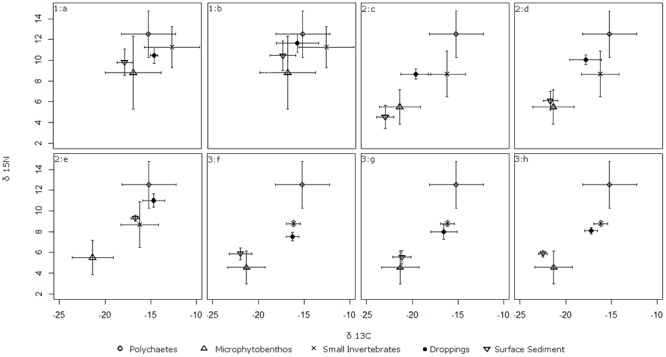
Mean isotope values of *δ* ^13^C and *δ* ^15^N (± standard deviation) for microphytobenthos, small invertebrates, large polychaetes, surface sediments (biofilm) and Western Sandpiper droppings. Samples were collected at eight study sites within the Fraser Estuary—Boundary Bay migratory stopover location, 23 April–7 May 2012. 1:a Mud Bay, 1:b Boundary Bay East, 2:c Roberts Bank Inter-causeway, 2:d Roberts Bank Brunswick Point, 2:e Roberts Bank Westham Island, 3:f Sturgeon Bank South, 3:g Sturgeon Bank North, and 3:h Sturgeon Bank Iona.

Kuwae et al. [10] estimated that microphytobenthos comprised 7–11% of surface sediments but was consumed by Western Sandpipers at a minimum proportion of 65%, suggesting Western Sandpipers may mechanically filter sediments to extract microphytobenthos when feeding. In contrast, studies of bill and tongue morphology and stomach content analysis suggest biofilm grazing may be unfiltered [15, 42]. Due to this uncertainty, we chose to use the isotopic values of surface sediments without adjustment for selective feeding on microphytobenthos, as a conservative estimate of biofilm contribution to Western Sandpiper diet. To ensure our models provided conservative estimates, microphytobenthos isotope values were plotted alongside those of droppings, small invertebrates, large polychaetes and surface sediments. Based on visual inspection, the *δ* ^13^C and *δ* ^15^N values of surface sediments and microphytobenthos were similar in both magnitude and direction from dropping samples (Fig 2). This ensured that models run using isotopic values of surface sediments, regardless of the proportion of microphytobenthos selectively extracted, provided a conservative estimate of biofilm feeding in relation to other prey sources.

#### Stable Isotope Mixing Models

We used the Bayesian mixing model SIAR [46] in R 2.15.2 [47], to estimate the proportion of biofilm in Western Sandpiper droppings at each site. We assumed diet-tissue isotopic discrimination between prey and droppings to be negligible [10]. Each model was run for 2 million iterations, with an initial discard of 50,000 iterations, and the remaining thinned by 15, resulting in 130,000 posterior draws to estimate the median and 95% credibility intervals of each prey type’s contribution to Western Sandpipers’ diet. We ran separate models for each of the eight sampling sites. Due to the high variance in polychaete C and N isotopic values, we chose to pool our polychaete samples across all sampling sites to prevent biases due to sites with low polychaete sample sizes (S2 Table). When the pooled values from our study (n = 12, *δ* ^13^C = -15.20 ± 2.95, *δ* ^15^N = 12.51 ± 2.23) were compared to those of Kuwae et al. [13] (n = 63, *δ* ^13^C = -15.43 ± 1.57, *δ* ^15^N = 12.01 ± 1.07) we found a difference of only 0.23 *δ* ^13^C and 0.5 *δ* ^15^N, less than 4%; suggesting that polychaete sample size did not significantly impact the interpretation of our results. Variation in small invertebrate isotopic values was comparatively low among sites and sample sizes were higher. We therefore pooled invertebrates within the three naturally delineated Wildlife Management Areas 1: Boundary Bay, 2: Roberts Bank and 3: Sturgeon Bank.

To measure the similarity of the estimates of biofilm consumption by Western Sandpipers between sites, we used the approach of Bond and Diamond [48], and calculated Bhattacharyya’s Coefficient (BC) for each pairwise comparison between sites. Mixing models produce results in a Dirichlet distribution (one whose components sum to 1), which can be compared using Bhattacharyya’s Coefficient [49–51]. We used the 130,000 draws from SIAR to calculate the median BC for each pair of sites. Like traditional measures of dietary overlap, such as Morisita’s Index [52–53], BC values range from 0 (no overlap) to 1 (complete overlap), and values of BC >0.60 are considered to represent significant overlap in diet [54–55].

### Sandpiper Counts

During daily sample collection for isotope analysis, numbers of Western Sandpiper and Dunlin in each site were estimated within three hours of the high tide. Experienced observers estimated the size of flocks, by tallying the number of groups of 100 or 500 birds within each flock. This method has an estimated error of approximately 20% [21]. The species composition of each flock was estimated by tallying the number of individuals of Western Sandpiper and Dunlin along visual transect lines through each flock, and multiplying the mean proportion of each species by the total flock size.

## Results

**Fig 3:**
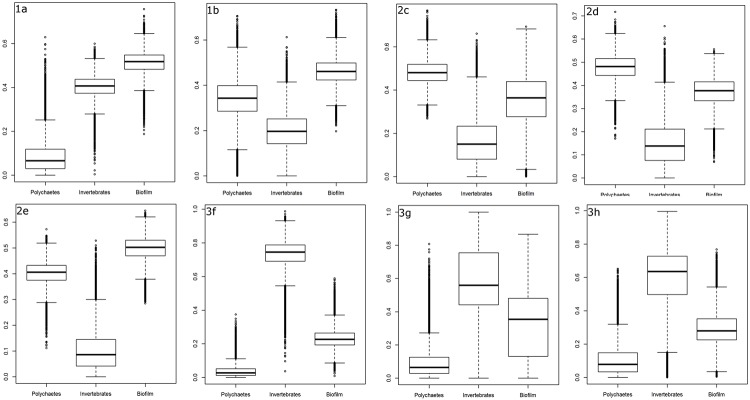
Proportion of large polychaetes, small invertebrates and surface sediment (biofilm) in Western Sandpiper diet based on dropping and prey samples collected during spring migration (23 April–7 May 2012). A three source mixing model of *δ* ^13^C and *δ* ^15^N was used for eight study sites within the Fraser Estuary—Boundary Bay migratory stopover location: 1:a Mud Bay, 1:b Boundary Bay East, 2:c Roberts Ban: Inter-causeway, 2:d Roberts Bank Brunswick Point, 2:e Roberts Bank Westham Island, 3:f Sturgeon Bank South, 3:g Sturgeon Bank North, and 3:h Sturgeon Bank Iona.

The proportion of biofilm in Western Sandpiper diet varied spatially with the highest proportions observed in Site 1:a Mud Bay, and Site 2:e Westham Island, and lowest proportions in Area 3: Sturgeon Bank (Table 1, Fig 3). Variation in diet constituents was relatively low for Area 1: Boundary Bay, Area 2: Roberts Bank and Site 3:f Sturgeon South (Table 1, Fig 3), and higher at Sites 3:g Sturgeon North and 3:h Sturgeon Iona where sample sizes were limited (S2 Table).

**Table 1:** Estimated contributions of surface sediments (biofilm), small invertebrates and large polychaetes in Western Sandpiper droppings across the Fraser Estuary—Boundary Bay migratory stopover location, 23 April—1 May 2012.

Site	Biofilm		Small Invertebrates		Polychaetes	
	Median	95% Cr.I.	Median	95% Cr.I.	Median	95% Cr.I.
1:a Mud Bay	51.7%	40.2–60.0%	40.7%	29.0–49.1%	6.7%	0.3–26.2%
1:b Boundary Bay East	46.1%	35.2–57.2%	19.7%	4.6–36.2%	34.2%	16.5–50.2%
2:c Roberts Bank Inter-causeway	36.4%	10.0–54.7%	15.0%	1.0–40.0%	48.1%	37.9–60.0%
2:d Roberts Bank Brunswick Point	37.7%	24.8–47.6%	13.7%	1.0–35.6%	48.2%	36.2–57.5%
2:e Roberts Bank Westham Island	50.2%	39.9–57.3%	8.6%	0.4–27.6%	40.6%	30.3–47.6%
3:f Sturgeon Bank South	22.8%	13.2–36.0%	74.4%	52.1–85.5%	2.8%	0.1–13.2%
3:g Sturgeon Bank North	35.9%	2.4–63.6%	55.9%	20.4–93.9%	6.4%	0.3–35.9%
3:h Sturgeon Bank Iona	27.9%	14.3–49.7%	63.7%	25.4–82.9%	7.8%	0.3–33.6%

Data are presented as the median of 130,000 posterior draws and 95% credibility intervals (Cr.I.), the Bayesian equivalent of confidence intervals

Diet composition of Western Sandpipers varied significantly between Areas 1: Boundary Bay, 2: Roberts Bank and 3: Sturgeon Bank, but not between sites within each of these three areas (Table 2). Diet composition in Site 1:a Mud Bay was significantly different from all three Area 2: Roberts Bank sites but not from all three Area 3: Sturgeon Bank sites (Table 2). Diet composition in Site 1:b Boundary Bay East was significantly different from all three Area 3: Sturgeon Bank sites but did not differ significantly from the three Area 2: Roberts Bank sites (Table 2). Diet composition at all three Area 3: Sturgeon Bank sites was significantly different from all three Area 2: Roberts Bank sites (Table 2). The spatial variation in diet composition (Table 1, Fig 3) suggests that Western Sandpipers in Site 1:a Mud Bay fed chiefly on biofilm and, to a lesser extent, on small invertebrates, whereas Western Sandpipers in Sites 1:b Boundary Bay East and Area 2 Roberts Bank fed chiefly on biofilm and large polychaetes, and those in Area 3: Sturgeon Bank fed chiefly on small invertebrates and a smaller amount of biofilm.

**Table 2:** Bhattacharyya’s Coefficient (BC) for pairwise comparisons of estimated diet of Western Sandpipers at study sites within the Fraser Estuary—Boundary Bay migratory stopover location.

	1:b	2:c	2:d	2:e	3:f	3:g	3:h Sturgeon Bank Iona
1:a Mud Bay	0.65	0.48	0.49	0.46	0.75	0.72	0.79
1:b Boundary Bay East	–	0.88	0.91	0.89	0.36	0.52	0.59
2:c Roberts Bank Inter-causeway		–	0.91	0.86	0.27	0.39	0.45
2:d Roberts Bank Brunswick Point			–	0.89	0.27	0.39	0.45
2:e Roberts Bank Westham Island				–	0.23	0.34	0.39
3:f Sturgeon Bank South					–	0.72	0.78
3:g Sturgeon Bank North						–	0.74

Higher BC indicates greater similarity in estimated diet, and BC >0.60 indicates significant similarity. Data are presented as the median BC of 130,000 iterations.

**Fig 4:**
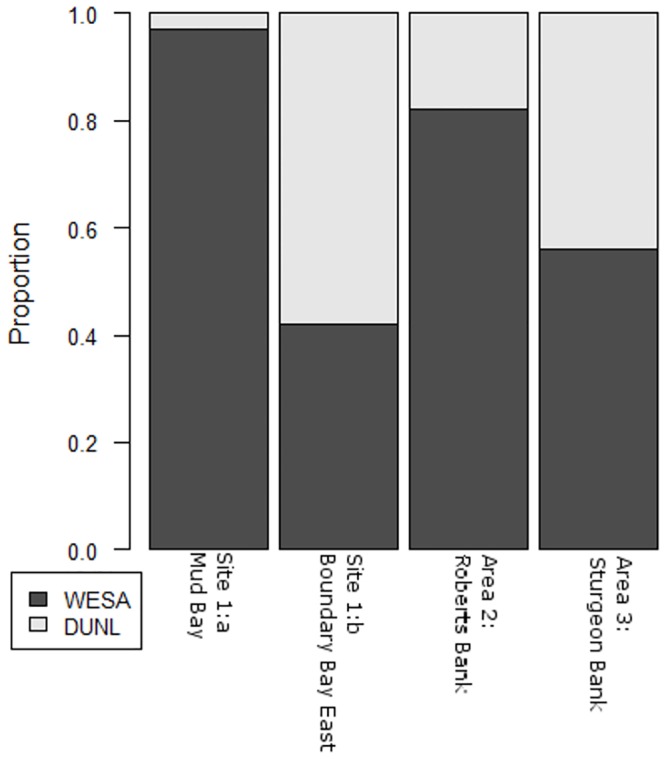
Average proportions of Western Sandpiper and Dunlin in all flocks of birds differentiated to species, in study sites or areas that exhibited significant differences in diet composition. Site 1a: Mud Bay (n = 1), Site 1b: Boundary Bay East (n = 6), Area 2: Roberts Bank (n = 8), and Area 3: Sturgeon Bank (n = 3).

Western Sandpiper and Dunlin were widely distributed in large numbers across the Fraser Estuary and Boundary Bay during the study period. The highest counts were recorded at Site 2:d Brunswick Point (Table 3), where 55% of all the Western Sandpipers detected were recorded; 17% were recorded in Area 3: Sturgeon Bank and 28% in Site 2:b Boundary Bay East. The proportion of Western Sandpiper to Dunlin at Area 2: Roberts Bank sites (0.82) was consistently higher than at Area 3: Sturgeon Bank sites (0.56) and Site 1:b Boundary Bay East (0.42). Site 1:a Mud Bay had the highest proportion of Western Sandpiper (0.97; Fig 4). In all areas, Western Sandpipers were observed using epifaunal feeding actions associated with biofilm consumption [10, 13] and regular movements of shorebirds between and within the eight study sites and the three larger areas were observed.

**Table 3:** Average total counts, and average estimated proportions of Western Sandpiper (WESA) and Dunlin (DUNL) from ratios in sample flocks within study sites in the Fraser Estuary—Boundary Bay migratory stopover location, 23 April–1 May 2012.

n	Area	WESA and DUNL Combined	WESA	DUNL	UNID
4	1:b Boundary Bay East	62125	15436	24414	22275
3	1:b Boundary Bay East (partial count)	11133	5167	5967	0
1	1:a Mud Bay	11300	11000	300	0
7	2:d Roberts Bank Brunswick Point	102100	73741	19502	8857
1	2:c Roberts Bank Inter-causeway	33000	30000	3000	0
1	2:e Roberts Bank Westham Island	5000	4500	500	0
1	3:h Sturgeon Bank Iona	7000	3900	3100	0
5	3:g Sturgeon Bank North	5300	4410	4590	1700
3	3:f Sturgeon Bank South	29300	0	0	29300

Counts where species ratios were not taken are reported as undifferentiated Western Sandpiper and Dunlin (UNDI) not identified to species (optical equipment, flocking behaviour or distance from observer precluded making species-specific estimate)

## Discussion

Biofilm was estimated to comprise 22.8% (95% credibility interval 13.2–36.0%) to 53% (95% credibility intervals 39.9–57.3%) of Western Sandpiper droppings, suggesting that it is an important dietary component throughout the stopover location. Given that droppings provide a conservative estimate of biofilm grazing [10] and that we assumed grazing was unfiltered, the values presented here likely under represent the true proportion of biofilm in Western Sandpiper diet. Our study followed an established protocol for Western Sandpiper dropping collection [10], and care was taken in collecting dropping samples, however we feel a study of the difference between droppings collected using this method and droppings collected directly from birds is warranted to test the assumption that droppings are not influenced when collected from mud- and sandflat surfaces.

Regular movements of Western Sandpiper and Dunlin between sites and areas suggest that birds have knowledge of food and habitat variation and make site selection decisions accordingly. The proportion of biofilm, small invertebrates and polychaete worms in the diet differed significantly between Site 1:a Mud Bay, Site 1:b Boundary Bay East, Area 2: Roberts Bank and Area 3: Sturgeon Bank. Two non-mutually exclusive hypotheses exist that may explain this diet heterogeneity: i) the diet composition in areas supporting the highest numbers of Western Sandpipers is optimal and the population is operating under an ideal free distribution [56], and ii) there are heterogeneous diet, behavioural or habitat requirements within sub-groups of the species and birds are selecting sites that meet their individual needs.

The sites with the highest proportion of biofilm in the diet, Area 2: Roberts Bank and Site 1:a Mud Bay either supported the greatest numbers of Western Sandpipers, or exhibited the highest proportions of Western Sandpipers to Dunlin, suggesting that site use was related to biofilm consumption. Analysis of bird density in comparison to biofilm abundance was not possible here, as density per unit area does not necessarily correlate to density per unit biofilm. The extent and density of biofilm varies spatially and temporally [14] yet efficient preliminary methods to quantify biofilm abundance in the Fraser Estuary, using infrared photography combined with sediment sampling, were only developed after the completion of our study [14]. When applied to Site 2:b Brunswick Point, these methods found that only 30% of the exposed intertidal area supported a rich microphytobenthos community [14]. Because biofilm acts as a major food source for the invertebrate communities on which shorebirds also prey [57], site selection will be influenced both by the presence of biofilm and by the invertebrate communities (meiofauna and macroinvertebrates) it supports. We collected samples from within 1 km of the high tide line/salt marsh edge during the falling tide. If birds move to areas further from shore during low tide periods of the tidal cycle, or in response to predation pressure, then diet constituents may differ. Physical substrate properties throughout the stopover site may also differentially impact the availability of prey resources [58] and therefore diet composition. These are important factors when considering potential development options, since hydrological changes may impact substrate composition, density or hardness.

Ideal free distribution theory assumes that all individuals have the same foraging preferences. Given that fattening rates of Western Sandpipers neither correlate to feeding strategy, nor vary within stopover sites [59] and Western Sandpipers exhibit a progressive downward shift in trophic position during northward migration, despite abundance of macrofaunal prey [6–7, 60], an ideal diet composition may not exist for this species as a whole, and optimal diet composition may be state-dependant [24–26]. A variety of factors may give rise to state-dependant foraging habits. Migrating shorebirds must balance body condition with predation risk: leaner birds utilize food-rich, but dangerous, habitats and heavier birds use safer areas with lower food availability [24, 28, 43]. Age and migratory stage differences in digestive physiology have been demonstrated for this species [61–63] and because of their large non-breeding range [17], some Western Sandpipers undertake longer migratory journeys then others. Western Sandpipers may also employ sex-specific feeding strategies [6, 26, 33, 64], leading to heterogeneous site use [65–67]. Female bills average 13% longer than males’ and male Western Sandpipers use epifaunal (surface) feeding associated with biofilm grazing more often than females, who use infaunal (probing) feeding, despite equivalent prey abundance [29]. Latitudinal separation in sex and size distribution across the non-breeding range [68–70] and site-based sex bias between habitat types within the same latitudinal band [71] are established strategies for this species. All these factors may lead to different utilization of food resources if different sites or areas are optimal for the feeding strategies of different sub-groups based on migratory distances, physiology, body condition, age and sex [7, 61].

Although we focused on spring migration, similar studies during the extended fall migratory period are needed in order to understand the implications of diet heterogeneity and biofilm feeding throughout the full life cycle of this species. Shorebirds have a generally high flexibility of invertebrate prey species choice, and previous management regimes have focused on maintaining wetland habitats and diverse invertebrate populations [72]. However, if shorebirds feed extensively on biofilm during northward migration, or throughout the entire migratory cycle, management strategies that maintain biofilm and invertebrate diversity may be required. Intra-guild competition for biofilm resources [13] between Western Sandpipers and the invertebrates on which they feed, and interactions between the spatial organizations of invertebrates, biofilms and shorebirds reinforce the need for this approach.

Biofilm feeding by vertebrates is a relatively recent discovery [10, 13]. Our results demonstrated the widespread use of multiple areas within a stopover site by Western Sandpipers, and confirmed that substantial biofilm consumption occurs in each of these areas. Our finding that Western Sandpiper diet composition varied significantly between sites concurs with a study of Semipalmated Sandpipers (*Calidris pusilla*) in the Bay of Fundy [16] and has potential conservation implications. Understanding whether heterogeneity in resource availability is necessary to support all sub-groups of a population during migratory stopover, and whether shorebirds demonstrate a preference for areas of high biofilm abundance and/or quality are important research priorities [26]. The potential consequences of habitat change, both within the Fraser Estuary—Boundary Bay system and at other major migratory stopover locations under similar development pressures, warrant a comprehensive study of the factors influencing shorebird site use to assess the relative importance of sites, to identify the potential impacts of infrastructure projects, and help guide responsible coastal development.

## Supporting Information

S1 Table Summary of secondary isotopic reference materials (SIRMs) used to calibrate unknowns and measure instrument precision. (DOCX)

S2 Table Numbers of samples collected for isotopic analysis in each of 8 study locations within the Fraser Estuary—Boundary Bay stopover location, British Columbia Canada, 23 April–7 May 2012. Dropping samples represent 10 droppings from different individuals pooled as one sample. Small invertebrates and polychaete samples represent a pool of all individuals captured at each excavation location.
(DOCX)
