# Supplementary material for: Biofilm Consumption and Variable Diet Composition of Western Sandpipers (Calidris mauri) during Migratory Stopover
Source: PLoS One. 2015 Apr 14;10(4):e0124164. doi: 10.1371/journal.pone.0124164 (PMC4397082; doi:10.1371/journal.pone.0124164)
Supplement: S1 Table — (DOCX) [file pone.0124164.s001.docx]

|  | **Accepted Value (mean ± SD)** | | **Within Runs (range of SDs)** | | | **Overall Among Runs (mean ± SD)** | | |
| --- | --- | --- | --- | --- | --- | --- | --- | --- |
| **SIRM** | ***δ*^13^C** | ***δ*^15^N** | ***δ*^13^C** | ***δ*^15^N** | ***n*** | ***δ*^13^C** | ***δ*^15^N** | ***n*** |
| **Biological SIRMs** |  |  |  |  |  |  |  |  |
| Casein protein | –26.68 ± 0.13 ‰ | +5.94 ± 0.08 ‰ | - | - |  | –27.30 ± 0.09 ‰ | +6.10 ± 0.07 ‰ | 3 |
| IAEA CH7 polyethylene foil | –32.15 ± 0.10 ‰ | - | - | - |  | –32.19 ± 0.15 ‰ | - | 8 |
| IAEA N2 ammonium sulfate | - | +20.3 ± 0.14 ‰ | - | - |  | - | +20.28 ± 0.14 ‰ | 8 |
| Acetanilide (batch 149699) | –31.59 ± 0.12 ‰ | –2.32 ± 0.23 ‰ | 0.04-0.24 ‰ | 0.01-0.22 ‰ | 8 | –31.68 ± 0.19 ‰ | –2.24 ± 0.29 ‰ | 50 |
| Bovine liver standard (BLS) | –18.8 ± 0.14 ‰ | +7.18 ± 0.17 ‰ | 0.05-0.25 ‰ | 0.06-0.13 ‰ | 5 | –18.75 ± 0.13 ‰ | +7.16 ± 0.14 ‰ | 16 |
| Nicotinamide | –34.52 ± 0.13 ‰ | –1.71 ± 0.10 ‰ | 0.04-0.14 ‰ | 0.04-0.19 ‰ | 5 | –34.51 ± 0.09 ‰ | –1.63 ± 0.20 ‰ | 18 |
| Small-mouth bass muscle (SMB-M) | –23.41 ± 0.18 ‰ | +12.31 ± 0.11 ‰ | 0.04-0.24 ‰ | 0.04-0.26 ‰ | 5 | –23.37 ± 0.12 ‰ | +12.35 ± 0.16 ‰ | 18 |
| **Sediment SIRMs** |  |  |  |  |  |  |  |  |
| NIST 1547 Peach leaf | –26.17 ± 0.08 ‰ | +1.94 ± 0.12 ‰ | 0.01-0.13 ‰ | 0.07-0.09 ‰ | 2 | –26.24 ± 0.08 ‰ | +2.04 ± 0.30 ‰ | 6 |
| Aquatic moss standard (AQM) | –35.08 ± 0.15 ‰ | +16.14 ± 0.07 ‰ | 0.07-0.19 ‰ | 0.05-0.24 ‰ | 4 | –35.03 ± 0.13 ‰ | +16.07 ± 0.15 ‰ | 10 |
| Corn meal standard (CMS) | –13.25 ± 0.11 ‰ | +4.42 ± 0.12 ‰ | 0.07-0.12 ‰ | 0.08-0.17 ‰ | 4 | –13.19 ± 0.07 ‰ | +4.52 ± 0.11 ‰ | 9 |
| Ephedra plant standard (EPS) | –30.96 ± 0.09 ‰ | +0.35 ± 0.12 ‰ | 0.08-0.12 ‰ | 0.09-0.17 ‰ | 3 | –31.08 ± 0.09 ‰ | +0.32 ± 0.15 ‰ | 9 |
| Spirulina standard (SPL) | –24.97 ± 0.12 ‰ | +12.94 ± 0.09 ‰ | 0.04-0.09 ‰ | 0.03-0.07 ‰ | 3 | –24.97 ± 0.09 ‰ | +12.95 ± 0.15 ‰ | 10 |

Accepted values and overall values among all runs are presented as mean ± SD (n = number of duplicates), within runs, data are presented as the range of SDs (n = number of runs where the SIRM was run more than once).
