# Supplementary material for: Biofilm Consumption and Variable Diet Composition of Western Sandpipers (Calidris mauri) during Migratory Stopover
Source: PLoS One. 2015 Apr 14;10(4):e0124164. doi: 10.1371/journal.pone.0124164 (PMC4397082; doi:10.1371/journal.pone.0124164)
Supplement: S2 Table — Dropping samples represent 10 droppings from different individuals pooled as one sample. Small invertebrates and polychaete samples represent a pool of all individuals captured at each excavation location. (DOCX) [file pone.0124164.s002.docx]

| Area | Droppings | Surface Sediment | Small Invertebrates | Polychaetes | Microphytobenthos |
| --- | --- | --- | --- | --- | --- |
| Mud Bay | 9 | 9 | 1 | 1 | 1 |
| Boundary Bay | 31 | 30 | 2 | 5 | 4 |
| Roberts Bank (Inter-causeway) | 15 | 15 | 1 | 1 | 2 |
| Roberts Bank (Brunswick Point) | 15 | 15 | 2 | 1 | 1 |
| Roberts Bank (Westham Island) | 19 | 19 | 1 | 1 | 2 |
| Sturgeon Bank South | 25 | 19 | 7 | 1 | 4 |
| Sturgeon Bank North | 7 | 7 | 2 | 1 | 1 |
| Sturgeon Bank Iona | 4 | 4 | 2 | 1 | 1 |
| Totals | 125 | 118 | 18 | 12 | 16 |
